# Xanthine Oxidase/Dehydrogenase Activity as a Source of Oxidative Stress in Prostate Cancer Tissue

**DOI:** 10.3390/diagnostics10090668

**Published:** 2020-09-03

**Authors:** Andrej Veljković, Jovan Hadži-Dokić, Dušan Sokolović, Dragoslav Bašić, Ljubinka Veličković-Janković, Marko Stojanović, Dejan Popović, Gordana Kocić

**Affiliations:** 1 Faculty of Medicine, University of Niš, 18 000 Niš, Serbia; dusantsokolovic@gmail.com (D.S.); kocicrg@yahoo.co.uk (G.K.); 2 Serbian Academy of Sciences and Arts, 11 000 Belgrade, Serbia; jovanhdj@gmail.com; 3 Clinical Center, 18000 Niš, Serbia; basicdr@gmail.com (D.B.); dravel@mts.rs (L.V.-J.); mimtroska@gmail.com (M.S.); dena.popovic@mts.rs (D.P.)

**Keywords:** prostate cancer, oxidative stress, xanthine oxidase

## Abstract

Prostate cancer (PC) is one of the most frequent malignancies. Better biomarkers are constantly wanted, such as those which can help with the prediction of cancer behavior. What is also needed is a marker which may serve as a possible therapeutic target. Oxidative stress (OS), which is a hallmark of cancer, is included in the pathogenesis and progression of PC. We have conducted the present study to determine whether xanthine oxidase/dehydrogenase activity is the source of OS in prostate tissue. We have also determined the concentration of TBA-reactive substances (TBARS) and advanced oxidation protein products (AOPP), as well as the activity of catalase. Xanthine oxidase (XO) activity is significantly higher (*p* < 0.001) in tumor tissue when compared to the control healthy tissue. The concentration of TBARS (*p* < 0.001) and AOPP (*p* < 0.05) are also higher in tumor tissue. Catalase has raised its activity (*p* < 0.05) versus the control. There is also a strong correlation between XO activity and prostate-specific antigen (PSA) levels in the serum. These results indicate a significant role of XO activity in OS in prostate carcinogenesis, and it could be a possible theranostic biomarker, which can be important for a better understanding of the disease, its evolution, and prognosis. A promising treatment may be using XO inhibitors such as allopurinol as adjuvant therapy.

## 1. Introduction

Prostate cancer (PC) is one of the most frequently diagnosed malignancies, the second among male population. Its incidence increases with age, especially in developed countries [1,2]. Even though the incidences of PC are growing rapidly, the mechanisms which result in the onset and development of carcinoma remain unclear. A considerable scientific interest has been placed on the molecular pathogenesis of PC. Better biomarkers are constantly sought, such as those which can help not only with diagnosis, but also with the prediction of cancer behavior, and the degree of malignancy. What is also needed is a marker which can be a possible therapeutic target.

The emerging evidence has shown that oxidative stress (OS), as a hallmark of cancer, is included in the pathogenesis and progression of PC, especially with the tumor metastasis and therapeutic resistance [3,4]. OS is caused by an imbalance between the production of reactive oxygen species (ROS) and their detoxification by the antioxidative system. When the level of ROS increases, they target cells, which leads to oxidative damage from the interaction of reactive oxygen with critical cellular macromolecules [5]. Oxidative stress can trigger pro-carcinogenic processes and is a key event in the initiation, invasion, and progression of PC. In prostate, OS related to age, precancerosis, and nutrition, as well as exposure to androgens, lead to pro-carcinogenic events [6,7]. It has been suggested that PC patients have a systemic imbalance in their OS/antioxidant status, when compared to healthy subjects or patients with Benign prostatic hyperplasia [8]. While a low increase in ROS levels promotes cell proliferation and differentiation, a high accumulation of radicals induces oxidative damage to lipids and proteins. ROS can also attack DNA directly and form mutagenic lesions [9,10]. ROS may also set off the formation of DNA adducts indirectly by initiating autocatalytic lipid peroxidation. Lipid peroxidation generates a large variety of potential genotoxic breakdown products, including alkoxy radicals, peroxyl radicals, and aldehydes, such as thiobarbituric acid reactive substances (TBARS) [11,12]. Lipid peroxidation is the most significant negative consequence of ROS generation. It causes irreversible damage to the function and structure of cell membrane. TBARS are the final products and indicators of lipid peroxidation level [13]. Several studies have shown that proteins are also changed by oxidants in cells and that oxidized proteins accumulate by age, oxidative stress, and in some types of cancer as the final and irreversible product of protein oxidation [14], especially advanced oxidation protein products (AOPP).

There are two major sources of cellular ROS. The major source in the mitochondria, responsible for fatty acid oxidation, is the family of NADPH oxidase enzymes. It transports electrons across biological membranes to generate superoxide O2^−^ by the reduction of oxygen [15]. Another cellular source of ROS is the peroxisomes. Owing to the presence and action of xanthine oxidase (XO), peroxisomal ROS consists predominantly of superoxide anion radicals [16]. Xanthine dehydrogenase (XDH)-mediated purine metabolic reactions produce ROS, including superoxide anion (O2^•−^) and hydrogen peroxide (H_2_O_2_) [17]. It is well known that xanthine oxidoreductase (XOD) is an enzyme present in two interconvertible forms, XDH and XO. Most researchers agree that dehydrogenase activity is converted to oxidase form, which produces hydrogen peroxide and superoxide. The process occurs through oxidation of sulfhydryl groups or by stimulated proteolysis. Both forms are capable of causing NADH oxidation with simultaneous formation of ROS [17,18]. Since the XO reaction produces ROS, along with uric acid, this enzyme represents the main source of ROS liberation in circulation.

It has been found that cancer cells are more susceptible to an acute increase in intracellular ROS levels than benign counterparts due to a significantly higher basal ROS level of XO [19]. It has not been clarified whether the activity of XO increases or declines in human cancers. In clinical practice, XO is the therapeutic target of drugs like allopurinol and febuxostat for gout or hyperuricemia [20].

Contrary to oxidants, there are several antioxidant mechanisms in cells operating through enzymatic reactions (e.g., superoxide dismutase, catalase, and peroxidase), and non-enzymatic small molecule antioxidants (vitamin E/C, glutathione, etc.) [21]. The most significant antioxidative activity belongs to enzymes which can decompose the ROS. Catalase (EC 1.11.1.6, hydrogen peroxide oxidoreductase) is a heme-containing tetramer, which catalyzes the decomposition of hydrogen peroxide into water and molecular oxygen. It is one of the primary antioxidative enzymes involved in the protection from oxidative stress [22]. A few studies have described the altered prooxidant–antioxidant status in the prostatic tissue of humans, rats, or permanent cell lines, but with paradoxical results [23,24].

Despite these controversial observations regarding XO activity and the role of OS in PC as a consequence of its activity, the clinico-pathological significance of XO activity in PC and the potential benefit as surrogate or theranostic biomarker have not been fully elucidated. We have conducted the present study to evaluate whether xanthine oxidase activity, lipid peroxidation, and activity of catalase may be developed as tissue biomarkers. Those biomarkers could identify patients with prostate cancer, predict the prognosis of the disease and include possible inhibitors of XO as adjuvant therapy. The side-effects of treatment of indolent tumors may cause increased morbidity as well as deteriorated quality of life with no improvement of the global survival, while treatment delay may lead to incurable disease [25]. The main objective of this study is to identify biomarkers of OS which could differentiate the severity of PC. The second objective is to delineate theranostic biomarkers, which may be used in the clinical practice as predictors of PC above and beyond the effects of prostate-specific antigen (PSA), even as potential therapy aimed at its inhibition.

## 2. Materials and Methods

We conducted the investigation at the Clinical Center in Niš, Serbia, from March 2018 until February 2020. Sixty men newly diagnosed with prostate cancer (from 62 to 73 years of age: mean 67.83 ± 3.22 SD), who had not undergone any previous treatment, were enrolled in this study. All subjects gave their informed consent for the inclusion before they participated in the study. The study was conducted complying with the Declaration of Helsinki, and the protocol was approved by the Ethics Committee of Clinical Center in Niš (Decision No. 27771/10, approved on 5 September 2017) day month year).

The criteria for exclusion from the study were the following: liver dysfunction, other malignancies, in-operability, previous chemotherapy, or radiation therapy heart failure or renal failure, smoking and oral antioxidant supplementation before the operation. Patients with gout, who were taking allopurinol were also excluded from the study. All prostate cancer patients were classified according to TNM classification. Characteristics of the study group are shown on Table 1.

Tissue specimens used for this study were collected by the pathologist, shortly after the resection of the carcinoma. Additionally, as a control, we collected the same amount of samples from the macroscopically unchanged prostate region farthest from the cancer. We also collected adjacent tissue, which surrounded the tumor with no macroscopic or pathological manifestations. Every specimen was examined by a pathologist to confirm that the appropriate part of the tissue was collected and that there were no cancer parts in the tissue which served as a control.

### 2.1. Preparation of Tissue Samples

All samples were placed in iced 0.15 mol/L NaCl solution, perfused with an isotonic solution to discard blood cells and other tissue residues. Further on, after the removal of fat, connective tissue, and major vessels, the tissue was cut into small pieces and washed with de-mineralized water to remove blood cells as much as possible and, subsequently, with 0.15 M phosphate-buffered (30 HIM) saline (pH7.5). We homogenized the tissue with a homogenizer with teflon pestle; made 10% homogenates and centrifuged them at 3000× *g* for 15 min. The supernatant was frozen at −80 °C and kept until assayed.

### 2.2. Biochemical Assays

#### 2.2.1. XOD and XO Activity

XOD and XO activity in the tissue homogenate were estimated by the amount of uric acid produced by using xanthine as substrate, in the presence of NADH (for XOD) or absence of NADH (for XO) when only molecular oxygen is an electron acceptor, for the fixed time interval. XOD and XO were measured in plasma according to the liberation of uric acid by using xanthine as substrate, in the presence of NADH (for XOD) or absence of NADH (for XO) when only molecular oxygen was electron acceptor [26]. The XDH activity was calculated by subtracting from XOD the XO activity. The molar extinction coefficient of 7.6 × 10^−3^ M cm^−1^ was used for this purpose [27]. XO and XDH activity was expressed as U/mg tissue protein in the homogenate.

#### 2.2.2. TBARS Concentration

We measured the TBARS concentration according to a slightly modified method previously described by Sahreen et al. [26]. The TBARS method was used to determine a low molecular weight of the aldehyde-malondialdehyde (MDA) which reacts with the thiobarbituric acid, forming a pink complex. The reaction mixture contained: 0.2 mL of the tissue homogenate, 0.2 mL of the ascorbic acid (100 mM), 0.58 mL of the potassium phosphate buffer (0.1 M; pH = 7.4), and 0.02 mL of the Ferric chloride-FeCl3 (100 mM). After being incubated for 60 min in a water bath at 37 °C, 1 mL of the CCl3COOH (10%) was added to the reaction assay. Afterwards, 1 mL of the TBA (0.67% dissolved in 0.1 M NaOH) was added to the reaction assay and heated for 30 min in a boiling water bath (100 °C). After cooling, 5 mL of the butanol-pyridine mixture (15:1) was added to the reaction mixture. After the centrifugation at 4000 rpm for 10 min, the absorbance of a clear supernatant layer was read at 535 nm against a butanol–pyridine mixture. The concentration of the TBARS was expressed in nmol/mg proteins.

#### 2.2.3. AOPP Concentration

The concentration of AOPP was determined by spectrophotometric technique according to the method of Witko et al. [28]. We diluted 200 microliters of supernatant in 1:5 in PBS; chloramine-T standard solutions were placed in wells of a 96-well microtiter plate, followed by 20 μL of acetic acid. Ten microliters of 1.16 M potassium iodide was added, followed by 20 μL of the glacial CH3COOH. The absorbance of the mixture was immediately read at 340 nm in a micro-plate reader against a blank containing all reagents. We calculated the concentration of the AOPP based on the standard curve of chloramine T (0–100 µmol/L) and expressed it in μmol/mg chloramine T.

#### 2.2.4. Catalase Activity

Catalase activity in tissues was determined using a slightly changed spectrophotometric method described by Nabavi et al. [29], based on the ability of catalase to dissolve the substrate (H_2_O_2_), whereby enzymatic reaction was stopped by the addition of ammonium molybdate. Enzyme activity was expressed in μmol/min/mg protein.

#### 2.2.5. Determination of Protein Concentration

The protein concentration in the tissue homogenate was determined according to the method previously described by Popović et al. [30]. The reaction mixture contained 10 µL of the diluted prostate homogenate and 150 µL of the reagent C (1 mL of 1% CuSO_4_, 1 mL of 2% potassium sodium tartrate, and 98 mL Na_2_CO_3_ dissolved in 0.1 M NaOH). After 30 min of incubation, 30 µL of the Folin reagent was added to the mixture (Folin–Ciocalteu reagent and water mixed in a ratio of 1:2). After 20 min, the absorbance was read at 550 nm, and the proteins concentration was expressed as mg protein/L.

### 2.3. Statistical Data Processing

The values of obtained parameters were expressed as X ± SD (mean value ± standard deviation), and as a box plot. The obtained results were assessed by the t-test by comparing the enzyme activity of mucosa with pathological manifestations or mucosa adjacent to tumor tissue with the activity of corresponding further healthy tissue as well as with the activity of tissue obtained from patients without any pathological manifestations. The association between any two continuous parameters was estimated using Pearson correlation test. The value of *p* < 0.05 was considered statistically significant.

## 3. Results

In the first graph, XOD, its dehydrogenase form (XDH) and oxidase form (XO) are represented. The data obtained after analysis are represented as mean value in control healthy tissue, tissue adjacent to cancer, but without pathohistological implications of the cancer process, as well as cancer tissue. The total XOD activity was significantly higher in tumor when compared to the control healthy tissue. The tissue adjacent to tumors also had a statistically higher activity when compared to healthy. The most significant rise was in XO expression, especially in tumor tissue. This rise was not due to conversion of HDX (Xanthine dehydrogenase) to XO, since the HDX activity was significantly lower in tumor versus healthy tissue.

In the next graph, the activity of XO is represented as a box plot. The data obtained after the analysis are represented as the mean and standard error.

The activity of XO significantly increased (*p* < 0.001) in tumor versus healthy control tissue. The adjacent tissue also had a higher activity (*p* < 0.001), but not as high as in the tumor tissue.

In the next set of experiments, the level of lipid peroxide is measured via TBARS concentration, and also protein oxidation products as AOPP in control healthy tissue, tissue adjacent to cancer, but without pathohistological implications of the cancer process, as well as cancer tissue. The data obtained after analysis are represented as the mean, standard error, and a vertical box plot.

The levels of TBARS increased significantly (*p* < 0.001) in tumor tissue when compared to the control healthy tissue. The adjacent tissue also had a higher activity, but with a lower statistical significance (*p* < 0.05). The level of AOPP statistically increased in tumor tissue when compared with control, but with a lower statistical significance (*p* < 0.05).

The activity of catalase in the tumor, the adjacent, and control healthy tissue was also analyzed.

Data obtained after the analysis are represented as the mean, standard error, and a vertical box plot.

The activity of catalase increased significantly (*p* < 0.05) in tumor versus healthy control tissue.

The association between activity of XO and PSA levels was determined using the Pearson correlation coefficient. A significant positive correlation (r = 0.6205 (*p* < 0.001)) was observed.

## 4. Discussion

Despite the available diagnostic methods for PC detection and prediction of its progressiveness and metastatic potential, an ideal non-invasive biomarker has not yet been discovered. The most commonly tested laboratory indicator of PC is a prostate-specific antigen (PSA), discovered over 50 years ago. It is now applied as the best serum marker for diagnosis and the monitoring of prostate cancer. However, it has certain limitations of specificity and sensitivity [31]. Although the level of serum PSA correlates with prostate cancer stage and tumor size, the widespread use of this biomarker has led to the discovery of numerous indolent cancers, which led to the problem of overdiagnosis and overtreatment [25]. Further development of diagnostic methods and evaluation of theranostic markers is essential. It would enable a better postoperative staging and a wider selection of most suitable therapeutic methods and post-treatment follow-up for individual patients. Considering that OS is involved in the pathogenesis of many human diseases, redox biomarkers are more common in clinical practice. The diagnostic importance of redox indicators has been evaluated in genetic diseases, metabolic disorders, and inflammatory diseases as well as in some types of cancer, such as gastric cancer, ovarian cancer, and melanoma [32,33,34].

In this study, we examined the usefulness of redox biomarkers to understand the mechanisms involved, and to identify biomarkers which can predict cancer invasiveness and targets for effective management of advanced prostate cancer with adjuvant therapy. The alterations in the human’s enzymology PC clearly distinguished it from the normal prostate tissue. To get a better understanding of OS inducers in PC, we paid more attention to investigating the interrelations between the XO activity as OS producer in human PC and the lipid peroxidation process and the antioxidative defense system evaluated via catalase activity. One of the most important mechanisms associated with carcinogenesis is OS. This phenomenon is defined as alterations in gene expression, cell metabolism, and cell homeostasis caused by overproduction of ROS and disturbances in antioxidant mechanisms [35]. It has been documented that OS causes genetic alterations, which can lead to PC [36]. In particular, the prostate which is exposed to androgenic with age, inflammatory, and nutritional insults, often suffers from insufficient capacity to neutralize OS [37]. We aimed to evaluate the importance of XO as an ROS producer since its activation has been implicated in ROS-dependent tissue damage under ischemia-reperfusion or hypoxia conditions [38,39].

**Figure 1:**
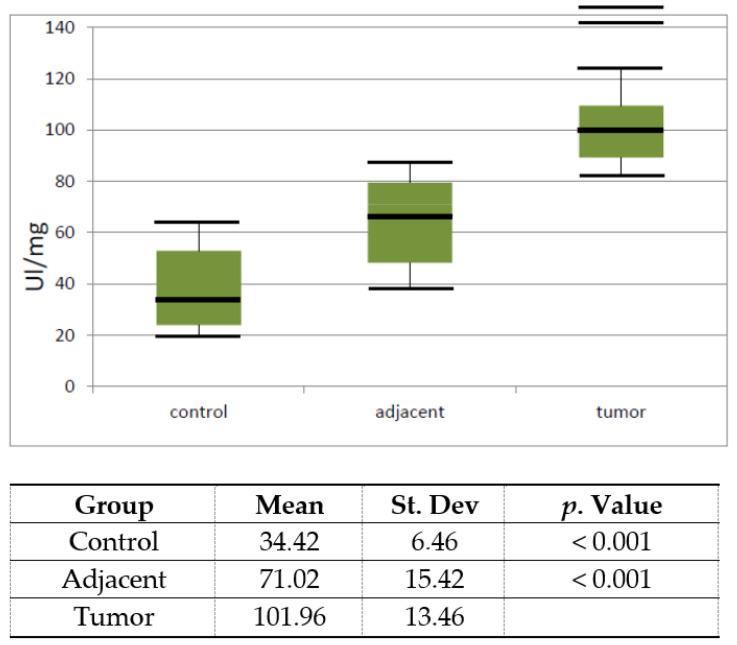
Vertical boxplot for Xanthine oxidase activity in control healthy tissue, tissue adjacent to tumor and tumor prostate tissue. The median is identified by a line inside the box, the boundaries of the box are interquartile range (IQR). Bars = entire range. Values more than 75th percentile are labelled as extreme and denoted as the horizontal lines beyond the bars.

**Figure 2:**
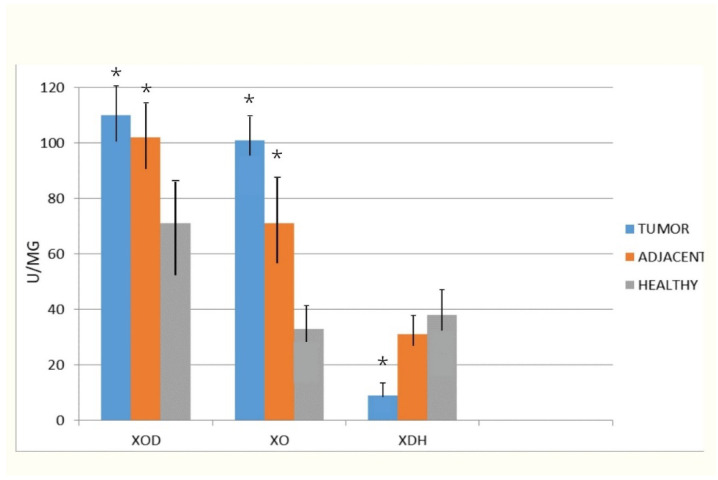
The activities of total Xanthine oxidoreductase (XOD), xanthine oxidase (XO), and xanthine dehydrogenase (XDH) in homogenates of investigated tissues. Xanthine oxidoreductase (XOD) and xanthine oxidase (XO) were measured in plasma according to the liberation of uric acid, in the presence of NADH (XOD) or absence of NADH (XO) when only molecular oxygen is an electron acceptor. The XDH activity was calculated by subtracting from XOD the XO activity * *p* < 0.001 compared with the healthy.

The results of our study indicated a more than twice higher activity of XO when compared to control tissue and tissue surrounding the tumor when compared to the control healthy tissue (Figure 1). Another important question concerns the mechanism involved in the upregulation of XO activity. We examined XDH activity to determine the percentage of conversion of the XDH to XO. So far, it has been reported that XOD activity is strictly regulated at the transcriptional and post-translational levels. Besides proteolytic and cysteine disulphide modifications, XDH/XO protein phosphorylation or even limited proteolysis were also reported previously [40]. We could relate increased XO activity to the conversion of the dehydrogenase form of XOD into the oxidase form, by reversible oxidation of thiol groups or by irreversible proteolytic attack caused by elevated peroxynitrite levels [41]. However, the results of our study suggested that only 8.91% of XO activity results from this conversion in tumor tissue (Figure 2). The expression of XOD protein is increased by various hormones, growth factors, inflammatory cytokines, irritation stimuli, and low oxygen tension [40]. Obviously, in prostate cancer, there are genetic changes which lead to high XO activity. The high XO activity in tumor tissue may also be explained by high levels of the xanthine, a substrate for its activity. In human cancers, studies on the significance of XOD expression are not widely reported. Some reports have showed that XDH protein expression and activity are much lower in tumor tissues compared to normal counterparts in gastrointestinal, breast, lung, bladder, and ovary tissues, in which XDH protein levels are normally expressed at a higher level [41]. Lower XDH levels in patient tumors are associated with a severe prognosis of cancer-specific survival in several types of cancers [42,43,44]. However, in accordance with our investigation, in liver cancer compared to noncancerous human liver tissues the activity of the dehydrogenase form of XOD decreased, but its oxidase form increased [45]. Significantly higher XOR levels were also observed in meningioma and astrocytoma when compared to a normal brain tissue [46]. Increased XOD activity was also reported in human laryngeal well-differentiated squamous cell carcinomas compared to the corresponding tumor-free adjacent tissues or normal laryngeal tissues [47].

**Figure 3:**
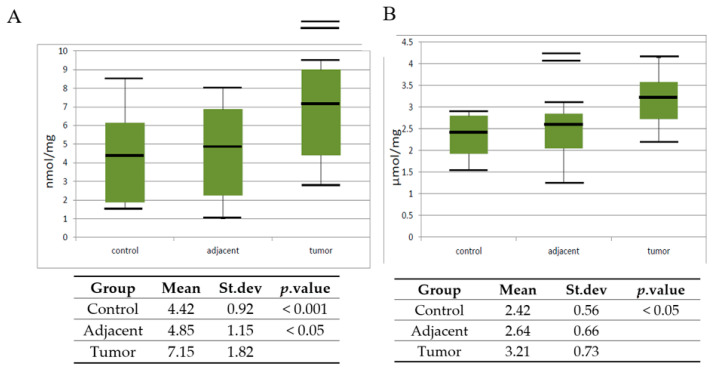
Vertical boxplot for (**A**) thiobarbituric acid reactive substances (TBARS) and (**B**) advanced oxidation protein products (AOPP) in control healthy tissue, tissue adjacent to tumor and tumor prostate tissue. The median is identified by a line inside the box, the boundaries of the box are interquartile range (IQR). Bars = entire range. Values more than 75th percentile are labelled as extreme and denoted as the horizontal lines beyond the bars.

ROS overproduction increases cancer progression and results in lipid peroxidation and protein oxidative damage [48]. To evolve the effect of high XO activity on elevated levels of OS, we determined the concentration of markers of oxidative changes on lipids and proteins. TBARS, the final product of lipid peroxidation, are a highly electrophilic molecule which reacts with cell nucleophiles to form DNA adducts and oligomers. Moreover, it reacts with several nucleic acids forming deoxyguanosine (dG), deoxyadenosine (dA), and deoxycytidine (dC) adducts [49]. It was shown that TBARS–DNA oxidation products had pro-mutagenic effects and induced mutations in oncogenes/tumor suppressor genes in human tumors [50]. In our study, the TBARS level was significantly higher in cancer tissue but with a low significance level (Figure 3A). Our findings are in accordance with the reports of Yilmaz et al. [51] on the elevated lipid peroxidation with antioxidant depletion in prostate cancer. However, Dogru-Abbasoglu et al. [52] have found no significant change in lipid peroxidation or antioxidant system parameters in the serum of patients with PC. The fact that lipid peroxidation is more prominent in erythrocytes and tumor tissue than in the serum of the patients could explain this diversity. Our results also showed increased levels of the protein oxidation in PC tissue versus control healthy tissue (Figure 3B). AOPP may be formed by the oxidation of a few amino acid side chains via the addition of aldehydes such as those generated from lipid peroxidation. Carbonyl proteins are an initial and reversible products from protein oxidation while Pande et al. [53] showed that AOPP levels were higher in PC patients as compared to the healthy control. Another case-control study reported no significant associations between PC risk or aggressiveness and protein oxidation products [54]. The results of our investigation indicate differences but they are not convincing, and there is no correlation between gradus of the carcinoma.

In mammalian cells, redox homeostasis is maintained at multiple levels between pro-oxidant and antioxidant factors. Although excessive ROS accumulation has been detected in human cancer cells, high levels of antioxidants also exist [55]. In accordance with this, we found a higher level of catalase activity in malignant tissue than in the healthy control and in the adjacent tissue. Jung et al. [23] have reported that there are no differences in the antioxidant enzymatic activities of prostatic epithelial cell cultures from benign and malign tissue. In another study, malignant epithelial cells in prostatic adenocarcinoma have been found to express lower levels of antioxidant enzymes than do benign prostatic epithelium [56] or almost no superoxide dismutase (SOD), glutathione peroxidase (GPX), and catalase (CAT) enzyme [57]. A few hypotheses could be offered to explain the depletion of the antioxidant defense: we could speculate that the circulating antioxidant enzymes might be used up in the attempt to counteract the enhanced lipid peroxidation in the tumor-affected tissue. Another speculation is that the enhanced lipid peroxidation occurs because of the insufficient power of a depleted antioxidant defense system for a prolonged time. Furthermore, as catalase is susceptible to oxidation by the oxidative reactive molecules and lipid peroxides, their substrates could deactivate those [58].

**Figure 4:**
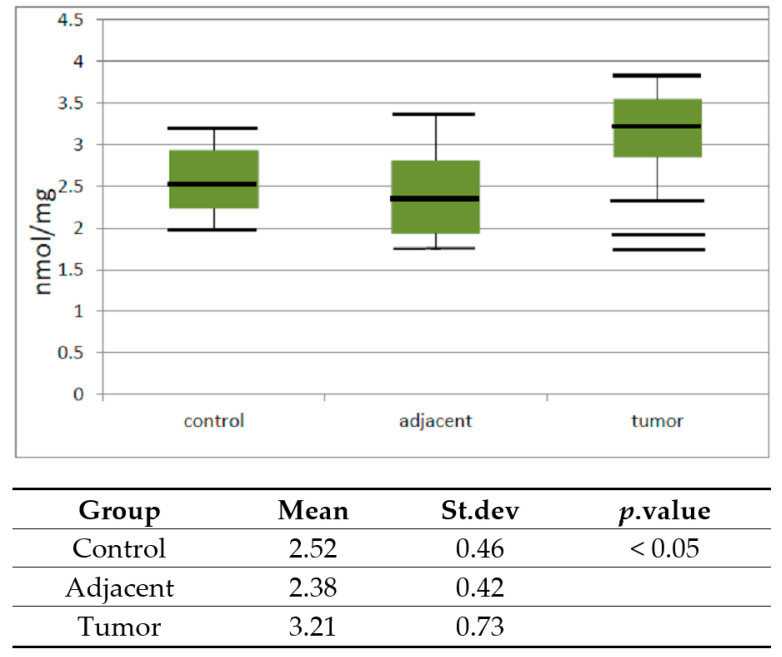
Vertical boxplot for catalase activity in control healthy tissue, tissue adjacent to tumor and tumor prostate tissue. The median is identified by a line inside the box, the boundaries of the box are interquartile range (IQR). Bars = entire range. Values more than 75th percentile are labelled as extreme and denoted as the horizontal lines beyond the bars.

The results of our research show an increase in catalase activity (Figure 4). The increase of the catalase activity can be explained as a compensatory measure. The basic role of catalase is the decomposition of H_2_O_2_ obtained in a process of OH radical dismutation. It has been demonstrated that PC tissue produces particularly large amounts of hydrogen peroxide (H_2_O_2_) [55]. The high concentration of H_2_O_2_ apparently raises the activity of catalase in prostate cancer tissue. These results reveal an alteration in the lipid peroxidation and protein oxidation with changes in the antioxidant defense system in prostate cancer patients. However, it remains unclear whether the alterations in the antioxidant status are the cause or consequence of the enhanced OS. It is certain that an altered prooxidant–antioxidant balance, despite the higher activity of catalase, may lead to increased oxidative damage, and consequently play an important role in prostate carcinogenesis. However, those changes are not significant and we do not propose them as a theranostic or surrogate biomarker. Our results are in line with the recommendations of previous studies, which have proven that the controlled application of antioxidative micronutrients failed to prevent PC [59]. Nevertheless, a recent study demonstrated that increased levels of oxidative damage and changes in the antioxidant defense system in high-risk subjects might have a possible link between oxidative stress and prostate cancer. The results of that study could be useful in risk stratification and in devising nomograms for treatment of prostate cancer [60].

**Figure 5:**
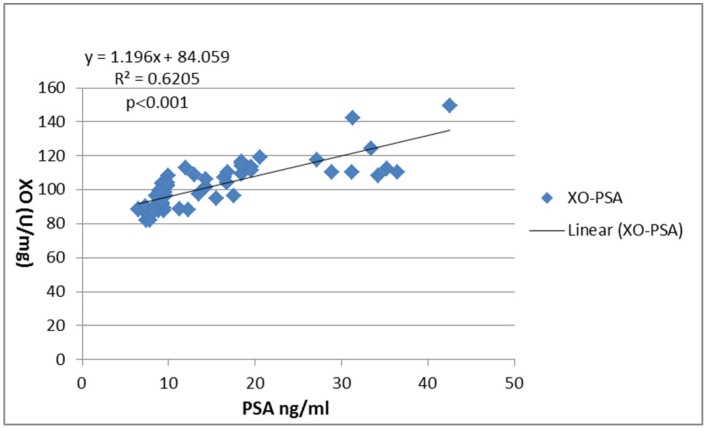
Correlation between XO activity in tumor tissue versus serum prostate-specific antigen (PSA). The correlation was estimated using the Pearson correlation coefficient (r) and illustrated using a scatter plot. *p*-value < 0.05 is considered significant.

Individualization of prevention strategies, using surrogate markers for prostatic oxidative stress, in correlation to the level of OS in tissue of PC could be a promising solution, especially because measurements of oxidative stress-related markers in the blood do not correlate to PC development [54]. However, the increased XO activity in patients with PC in our study suggests that it points to the role of this parameter as a marker of the disease evolution and suggests that it may affect the course of the disease. It is even in strong correlation to PSA values from the serum of the patients (Figure 5, Appendix A Table A1). The explosion of XO-mediated ROS in cancerous tissues may be caused by a large increase in substrate formation, which occurs due to rapid nucleotide production during the tumor growth process. The activity of xanthine XO rather than XDH also leads to the formation of hydrogen peroxide and hydroxyl radicals. Stimulated ROS generation is potentially responsible for cell membrane disruption and lipid peroxidation and leads to tissue and/or organ damage. Although further studies in PC patients are required, this may suggest that we can use the XO in these tissues as a surrogate marker for the individualization of PC prevention and therapeutics. Further research should investigate whether the XO as a biomarker could be used as differential diagnostic and prognostic tools in PC and BPH. They could help to improve the sensitivity and specificity of the existing detection techniques. The improved risk stratification and outcome prediction would enhance the physician’s ability to counsel patients about the treatment options and the associated risk and benefits. Facilitating the choice of appropriate treatment could improve the PC patients’ survival rate. A recently published article has shown that XO inhibition can suppress cell migration and metastasis of breast cancer [61] so that the use of allopurinol as adjuvant therapy in prostate cancer could be a promising treatment.

With regard to all the results, the limitations of our study should be mentioned. The evaluated redox biomarkers can only be used after the elimination of other oxidative stress-related disorders. On the other hand, the evident advantage of our study is the investigation of the enzyme in tissue samples collected from the patients with PC without any accompanying disease. We believe that XO as a potential biomarker could become a segment of non-invasive diagnostics. Namely, samples are collected from prostate with tumor after it has been removed, so that it does not represent a risk to the patient.

Another limitation of the study is whether the activity of XO in serum of the patients can be a significant marker. At the same, we should determine whether the high activity of this enzyme originates from tumor cells, since the prostate specimens used in this study, besides tumor cells, contain stromal cells. In follow up experiments, we can determine the activity of XO in the serum of the patients. However in that case we cannot determine the source of XO activity, so we should measure the XO activity is some of the cell lines of PC. Such experiments can be performed in comparison to the activity of the enzyme in stromal cells with the aim to determine the source of high activity of XO. However, a high activity in cell lines does not necessarily indicate a high activity in the cancer. In vivo, many activators and inhibitors can be found, so that the most relevant phenomenon is the activity in the cancer tissue. Further research of this sort goes beyond the limits of this study, the objective of which is to determine the existence of a potential cellular biomarker in the tissue. This biomarker can also serve as a potential target of adjunctive therapy with patients who exhibit a high activity of the enzyme.

Our experiment is also a starting point for further clinical trials which assess the diagnostic utility of redox biomarkers in a larger population of prostate cancers.

## 5. Conclusions

Based on the results of this research, we can conclude that oxidative stress which caused lipid peroxidation and protein oxidation, as well as the changes in catalase activity, might be included in prostate cancer pathogenesis. One of the possible causes of oxidative stress could be a high XO activity. The simplicity of measuring XO activity asserts the usefulness of this enzyme as theranostic biomarker together with the already known clinical-pathological findings, which can be significant in a better understanding of the disease, evolution, and prognosis. Using XO inhibitors such as allopurinol as adjuvant therapy could be a promising treatment.

## Figures and Tables

**Table 1:** Characteristics of the study group.

Parameter	*n* %
Age	
<70	36 (60%)
>70	24 (40%)
Stage at diagnosis	
II	38 (63%)
III	22 (37%)
Gradus	
2	31 (52%)
3	22 (37%)
4	7 (11%)
pN—lymph node metastasis	
No	25 (42%)
Nx	35 (58%)
pM-distant metastasis	
Mo	28 (47%)
Mx	32 (52%)
Gleason score	
6	15 (25%)
7	36 (60%)
8	9 (15%)
PSA level	
<10	31 (52%)
>10	29 (48%)

## Appendix A

**Table A1:** XO activity case versus the matched PSA level, Gleason scores, TNM stages, and ages.

XO Activity	PSA Values	TNM Stages	Gleason Score	Age
104.05	9.34	T2NoMo	7	72
109.25	18.45	T3NxMx	7	68
88.92	8.34	T2NoMo	6	66
107.22	16.45	T2NxMx	7	73
112.67	11.98	T3NoMx	7	64
102.33	9.45	T2NoMo	7	65
124.05	33.45	T3NxMx	8	68
116.44	18.45	T2NoMx	8	72
108.08	9.98	T2NxMo	7	69
82.96	7.67	T2NoMo	7	70
81.92	7.45	T3NxMx	7	64
88.34	6.56	T2NoMo	6	71
142.09	24.93	T3NxMx	7	70
87.6	9.45	T3NxMx	8	66
98.34	9.29	T2NoMx	7	69
119.02	20.54	T3NxMx	8	62
98.25	9.45	T2NoMo	7	72
108.8	13.02	T3NxMx	7	67
81.88	7.87	T2NoMo	7	69
113.72	18.45	T2NxMo	7	70
86.34	7.45	T2NoMo	7	67
112.34	18.89	T2NxMx	6	71
89.45	9.42	T3NoMx	6	64
91.41	9.25	T2NoMo	7	68
96.33	9.65	T3NoMx	7	65
87.42	8.87	T2NxMx	7	67
110.34	28.86	T3NoMx	7	73
94.76	15.54	T2NxMo	6	70
149.67	42.56	T3NoMx	7	68
88.88	6.54	T2NxMo	6	62
89.98	7.29	T2NoMx	7	65
96.45	17.56	T2NxMo	7	63
108.45	34.25	T3NxMx	8	65
88.33	12.25	T3NxMx	6	73
110.45	31.25	T3NxMx	8	70
88.78	11.25	T2NxMo	6	65
97.42	13.54	T2NoMo	7	64
117.45	27.17	T2NxMo	6	70
110.45	36.45	T3NxMx	6	64
104.34	9.87	T2NoMo	7	72
102.24	9.85	T2NxMo	6	67
113.45	19.56	T3NxMx	8	63
89.34	8.34	T2NxMo	7	72
92.34	9.24	T2NoMo	7	71
103.45	9.26	T2NxMo	7	66
111.45	19.56	T2NxMo	8	67
96.44	8.58	T2NxMx	7	63
88.87	8.42	T3NoMx	6	67
101.78	14.35	T3NxMx	7	62
104.23	16.75	T2NoMx	7	72
89.34	8.25	T2NxMo	7	68
98.34	9.56	T2NxMo	8	70
110.34	16.84	T3NxMx	7	68
106.45	14.36	T2NoMo	7	66
86.45	8.25	T2NxMo	6	72
98.34	9.56	T2NxMo	6	71
112.34	35.24	T3NoMx	7	67
102.74	9.89	T2NxMx	6	70
99.34	8.95	T2NxMx	7	64
115.45	18.45	T3NoMx	7	71
